# A novel tDCS control condition using optimized anesthetic gel to block peripheral nerve input

**DOI:** 10.3389/fneur.2022.1049409

**Published:** 2022-11-14

**Authors:** Silke Kerstens, Jean-Jacques Orban de Xivry, Myles Mc Laughlin

**Affiliations:** ^1^Research Group Experimental Oto-Rhino-Laryngology, Department of Neurosciences, Leuven Brain Institute, KU Leuven, Leuven, Belgium; ^2^Movement Control and Neuroplasticity Research Group, Department of Movement Sciences, The Leuven Brain Institute, KU Leuven, Leuven, Belgium

**Keywords:** transcranial direct current stimulation (tDCS), transcranial electric stimulation (TES), peripheral nerve input, topical anesthetics, BL10, tDCS control condition

## Abstract

**Background:**

Recent studies indicate that some transcranial direct current stimulation (tDCS) effects may be caused by indirect stimulation of peripheral nerves in the scalp rather than the electric field in the brain. To address this, we developed a novel tDCS control condition in which peripheral input is blocked using topical anesthetics. We developed a compounded anesthetic gel containing benzocaine and lidocaine (BL10) that blocks peripheral input during tDCS.

**Methods:**

In a blinded randomized cross-over study of 18 healthy volunteers (M/F), we compared the gel's efficacy to EMLA and an inert placebo gel. Subjects used a visual analog scale (VAS) to rate the stimulation sensation in the scalp produced by 10 s of 2 mA tDCS every 2 min during 1 h. In an additional *in-vitro* experiment, the effect of a DC current on gel resistivity and temperature was investigated.

**Results:**

Both the BL10 and EMLA gel, lowered the stimulation sensations compared to the placebo gel. The BL10 gel showed a tendency to work faster than the EMLA gel with reported sensations for the BL10 gel being lower than for EMLA for the first 30 min. The DC current caused a drastic increase in gel resistivity for the EMLA gel, while it did not affect gel resistivity for the BL10 and placebo gel, nor did it affect gel temperature.

**Conclusions:**

Topical anesthetics reduce stimulation sensations by blocking peripheral nerve input during tDCS. The BL10 gel tends to work faster and is more electrically stable than EMLA gel.

**Clinical trial registration:**

The study is registered at ClinicalTrials.gov with name “Understanding the Neural Mechanisms Behind tDCS” and number NCT04577677.

## Graphical Abstract

Effectiveness of topical anesthetics to block peripheral nerve input during tDCS. When tDCS is applied, peripheral nerves in the scalp are exposed to high electric fields that can reach 20 V/m. These electric fields are strong enough to stimulate cervical and cranial nerves (green) that innervate the scalp (beige). Stimulation of these peripheral nerves may indirectly contribute to observed tDCS effects. To investigate this, we developed a novel tDCS control condition in which peripheral nerve input is blocked using topical anesthetics. We optimized a compounded anesthetic gel containing 5% benzocaine and 5% lidocaine (BL10) and compared its effectiveness to reduce stimulation sensations during tDCS to EMLA, a commercially available anesthetic gel, and an inert placebo gel. Our results indicated that both anesthetic gels, BL10 and EMLA, significantly reduced the stimulation sensation during tDCS compared to the placebo gel. However, the BL10 gel tended to work faster than the EMLA gel as its stimulation sensation was immediately low after gel application. For the EMLA gel, subjects indicated a higher stimulation sensation compared to the BL10 gel in the beginning of the experiment, but the stimulation sensation decreased to a similar level after half an hour. The effectiveness of the topical anesthetics to block peripheral nerve input during tDCS is illustrated in the figure using shades of green for the cervical and cranial nerves in the scalp. When the placebo gel is applied, the high electric field in the scalp stimulates the peripheral nerves (dark green). The application of topical anesthetics underneath the stimulation electrodes effectively blocks peripheral nerve input during tDCS for the BL10 (light green) and the EMLA gel (green). 
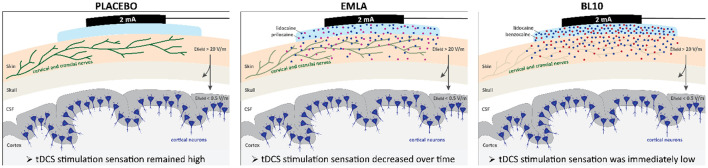

## Introduction

Transcranial direct current stimulation (tDCS) is a non-invasive neuromodulation method that is widely used by neuroscientists, neurologists and psychiatrists to modulate brain function. tDCS can modulate neurophysiological (1), physiological (2) and behavioral responses (3). For example, tDCS can improve memory (4), motor skill acquisition (5) and motor sequence learning (6). Currently, a wide range of clinical applications are being investigated, including tDCS treatments for improved motor function in elderly (7), improved cognitive function (8), stroke recovery (9), addiction (10), depression (11), epilepsy (12), and Alzheimer's disease (13).

The neurophysiological mechanism through which tDCS mediates these effects is debated (14). It has been generally accepted that tDCS modulates cortical excitability *via* the electric field in the brain causing subthreshold shifts in the resting membrane potential of cortical neurons (15, 16). Interestingly, a number of recent studies have shown that most of the applied current is actually shunted by the highly conductive scalp, leaving only a fraction of the applied current to reach the brain (2, 17). This means that during tDCS peripheral nerves in the scalp are exposed to high electric field strengths that can reach 20 V/m (17, 18). These fields are strong enough to initiate action potentials causing co-stimulation of the nerves in the scalp which may indirectly contribute to the observed tDCS effects (2, 14).

The scalp is mainly innervated by the trigeminal nerve (5^th^ cranial nerve), consisting of three branches: the ophthalmic nerve V1, maxillary nerve V2 and mandibular nerve V3. The dorsal part of the scalp is innervated by the occipital nerve (2^nd^ cervical spinal nerve), arising between the first and second cervical vertebrae, where it splits into the greater occipital nerve and the lesser occipital nerve (19). During tDCS, electrical activation of these nerves results in information ascending *via* the brainstem to the somatosensory cortex where it is perceived as the frequently reported tingling or itching sensation (20). In addition to the somatosensory input, the trigeminal and occipital nerves give input to other brain regions, including brainstem and limbic structures such as the locus coeruleus (LC), amygdala and hippocampus (21, 22). The LC is part of the reticular formation in the brainstem and is a key nucleus of the sympathetic nervous system controlling the synthesis and release of norepinephrine throughout the brain (23–27). This indirect pathway of tDCS may contribute to some of the observed tDCS effects. However, because the standard control condition used in all tDCS experiments is the sham condition in which current is simply switched off, the tDCS field currently lacks a robust control condition to carefully separate effects caused by the electric field in the brain from those caused by co-stimulation of nerves in the scalp.

**Figure 1:**
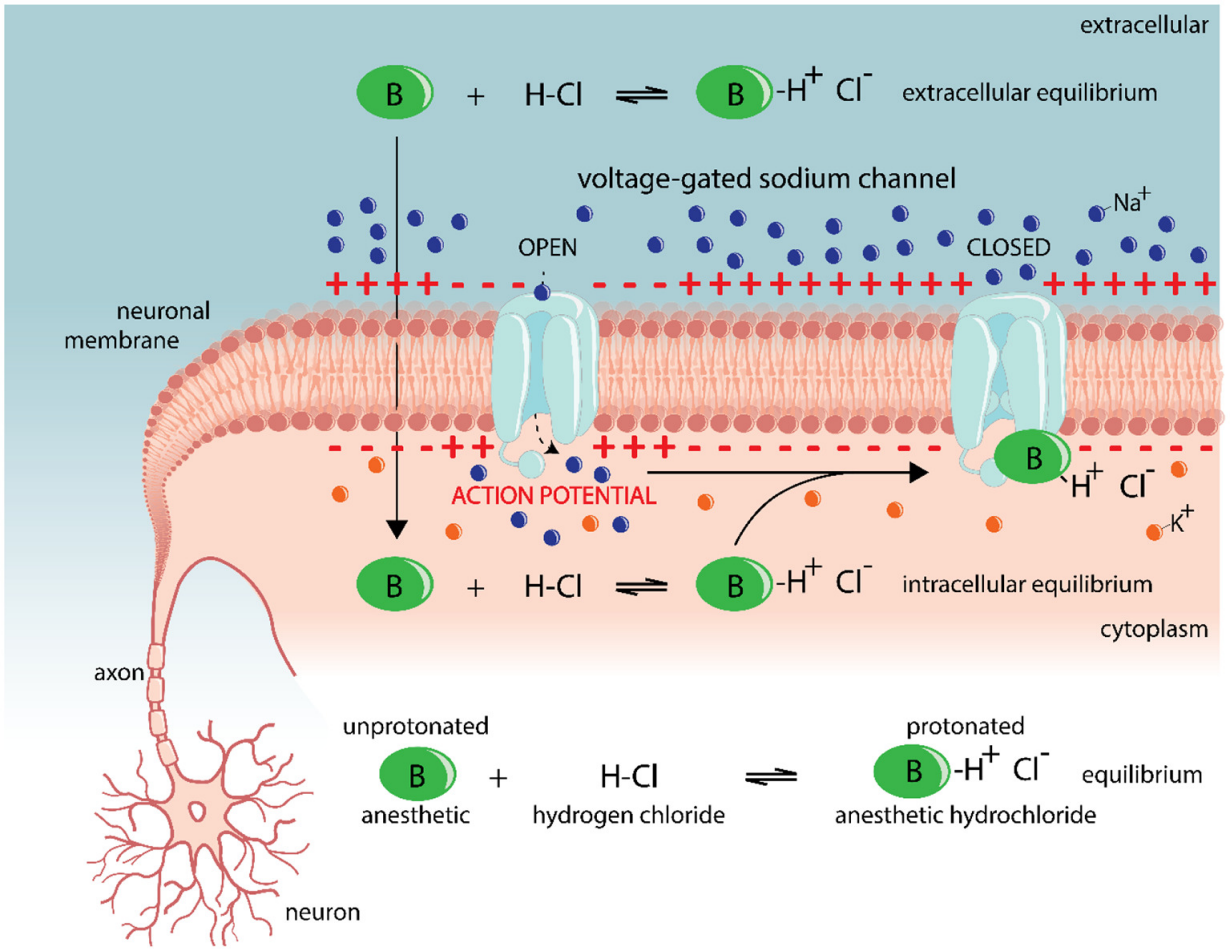
Mechanism of local anesthetics on voltage-gated sodium channels in neuronal cell membrane. Local anesthetics (green) pass through the cell membrane and then bind on voltage-gated sodium channels (light blue) on the inside of the cell membrane and block sodium-influx (dark blue) into the cell (28). Anesthetics are usually administered as a hydrochloride salt, a protonated form (BH+ Cl-) that is more water-soluble. However, the reaction is reversible and both forms, protonated and unprotonated, are in equilibrium with the relative amounts depending on the environmental pH. If the environmental pH is equal to the dissociation constant (pKa) of the anesthetic, usually between 8 < pKa < 9, both forms are present in equimolar amounts. In more basic environments like the extracellular space, the unprotonated form is favored while in slightly acidic environments such as in the cytoplasm, the protonated form is more present. The unprotonated form can easily cross the cell membrane, increasing the concentration on the inside of the cell. Once in the cytoplasm, it quickly transfers to the protonated form, which is charged and therefore unlikely to cross the membrane. This process of favoring the anesthetics to remain in the cell is referred to as “ion trapping.” In the cell, the protonated form of the anesthetic binds to the voltage-gated sodium channels, upon which it either physically blocks conductance through the channel, stabilizes the inactivated state of the channel or interferes with the channel gating machinery (28), depending on the type of the local anesthetic. As a result, the sodium flux into the cell is interrupted and the action potential (red) can no longer be generated or conducted along the cell membrane. In this way, anesthetics can block signal transduction in neuronal cells.

It has previously been shown that topical anesthetics can reduce peripheral nerve co-stimulation during transcranial alternating current (tACS) stimulation (17). Topical anesthetics reversibly prevent the induction or propagation of actions potentials in nerves (28), which can temporarily reduce peripheral nerve input. The working mechanism of topical anesthetics on neuronal cells is explained in more detail in Figure 1. In short, topical anesthetics diffuse into the cell and bind to voltage-gated sodium channels on the inside of the cell membrane. They can physically block conductance through the channel, stabilize the inactivated state of the channel or interfere with the channel gating machinery, depending on the type of the local anesthetic (28). The effectiveness of a topical anesthetic can be predicted based on its properties. The potency of a topical anesthetic is correlated with its affinity for the receptor site on the ion channel and its lipid solubility. Anesthetics with a high lipid solubility diffuse more easily through the cell membrane and can therefore reach its binding site more easily. However, the time of onset of an anesthetic is mainly determined by its dissociation constant (pKa). Anesthetics are usually administered as a hydrochloride salt, a protonated form (BH+ Cl-) that is more water-soluble. After administration, both forms, protonated (BH+) and unprotonated (B), exist in equilibrium in the extracellular space with the relative amounts depending on the pKa-value of the anesthetic and the environmental pH. The pKa of most anesthetics is between 8 < pKa < 9, which is higher than the physiologic pH = 7.4, meaning a greater proportion the molecules exists in the protonated form (BH+). For anesthetics with a lower pKa-value the equilibrium shifts in favor of the unprotonated (B) form, resulting in a higher diffusion rate across the cell membrane and therefore a shorter onset time (29).

In this study, we performed two experiments to investigate the efficacy of two topical anesthetics at minimizing peripheral nerve co-stimulation during tDCS. We designed a compounded anesthetic gel, consisting of 5% lidocaine and 5% benzocaine in a carbomer gel and compared this to the standard commercially available EMLA gel consisting of 2.5% lidocaine and 2.5% prilocaine and a placebo gel. Benzocaine has a very low dissociation constant pKa _benzocaine_ = 3.5 compared to the anesthetics in the EMLA gel, lidocaine pKa _lidocaine_ = 7.7 and prilocaine pKa _prilocaine_ = 7.7 (30). Hence, the BL10 gel containing benzocaine is expected to diffuse faster into the skin and therefore work faster (31). The control condition was an inherent placebo carbomer gel containing no anesthetics. In Experiment 1 we compared the effect of the gels at reducing tDCS stimulation sensations in the scalp in healthy volunteers, while in Experiment 2 we performed *in-vitro* measurements to assess gel resistivity and temperature changes during tDCS.

## Methods and materials

### Experiment 1

#### Anesthetic gels

The compounded anesthetic gel (BL10) consisted of 5% benzocaine and 5% lidocaine HCl in a water-based carbomer gel and was made by a compounding pharmacist. Standard EMLA gel (AstraZeneca, Cambridge, England) was used which contains 2.5% lidocaine and 2.5% prilocaine in an oil-based gel. As a control condition, an inert placebo gel was used consisting of a carbomer gel with no active components.

#### Ethical approval

The study was approved by the UZ/KU Leuven Research Ethical Committee (S63709) and conformed to the ethical standards described the Declaration of Helsinki on ethical principles for medical research involving human subjects. All experiments were performed in accordance with relevant guidelines and regulations. The study is registered in ClinicalTrials.gov (NCT04577677). Written informed consent was obtained from all subjects before inclusion.

#### Study design

A single-blinded randomized cross-over design was used to compare the effectiveness of the anesthetic gels at reducing skin sensations during tDCS, meaning the subjects were blinded to the gels. Each subject participated in three sessions, using a different gel in each session. The sessions were scheduled four to seven days apart.

#### Subjects

Eighteen healthy subjects participated in the study: ten females and eight males with age 24.8 ± 2.8. Standard tDCS exclusion criteria were used. All subjects were right-handed and had no history of migraine, addiction, epilepsy, or another neurological or psychiatric disorder. Other exclusion criteria were: metallic implants or implanted electrical devices, intake of drugs that affect the nervous system and pregnancy. In addition, subjects were asked to restrain from caffeine, alcohol, nicotine or other stimulants.

#### Transcranial direct current stimulation

tDCS was applied over the left primary motor cortex (M1) in which the anodal tDCS electrode was positioned over the left M1, on C3 according to the international 10–20 system. The cathodal reference electrode was placed on the contralateral supraorbital position, over FP2 according to the 10–20 system. A neuroConn DC-STIMULATOR PLUS (neurocare Group, Munich, Germany) was used to apply a current of 2 mA over Signa gel-filled cup electrodes with a surface area of 11.3 cm^2^ per electrode, held in place using an EEG cap.

#### Protocol

**Figure 2:**
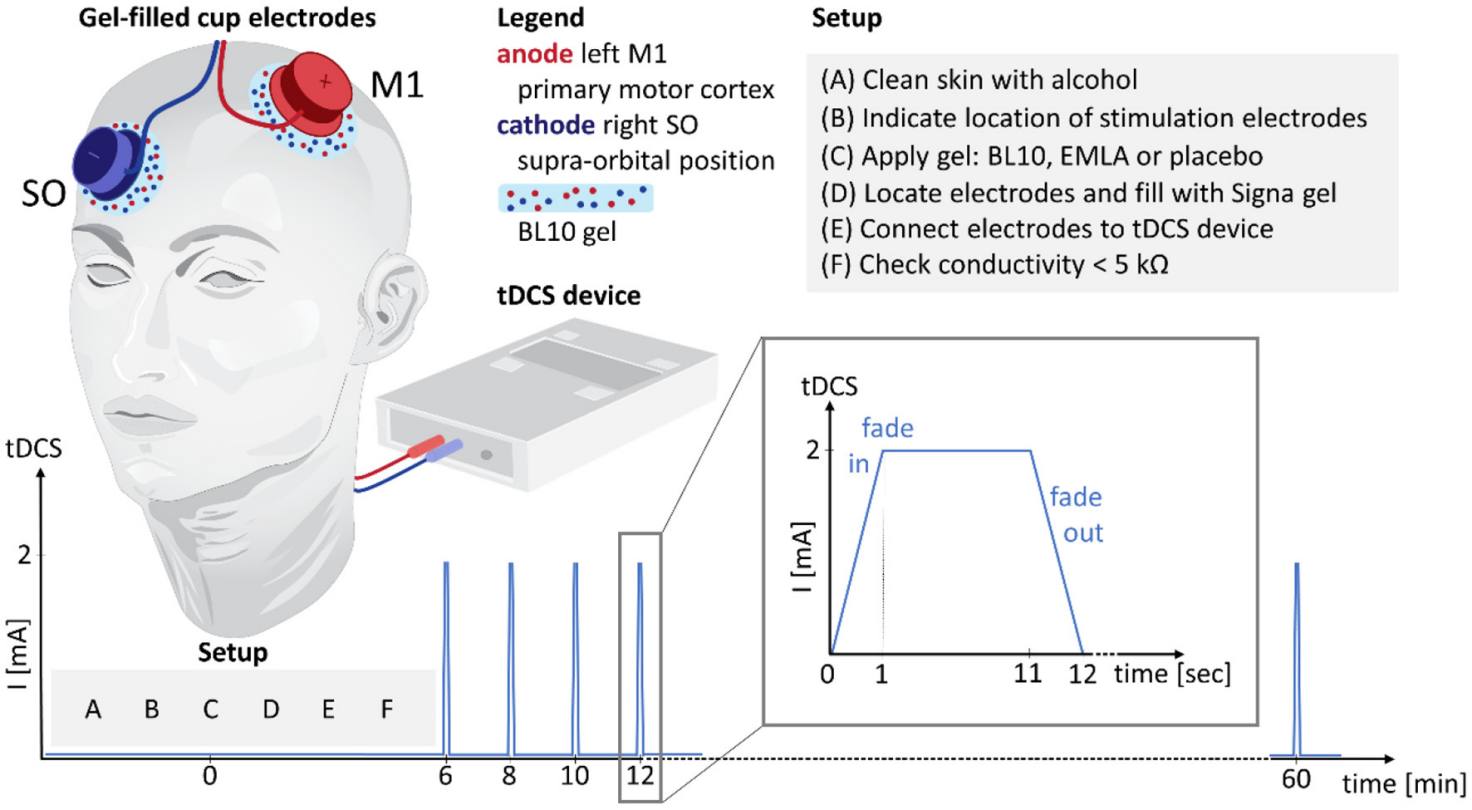
Protocol. Anodal tDCS was applied over the left primary motor cortex (M1) with the reference electrode in the contralateral supraorbital position. In the setup, the skin was cleaned with alcohol (A), the locations of the stimulation electrodes were indicated on the skin (B), 4.8 ml of anesthetic or placebo gel was applied under each electrode (C), the stimulation electrodes were located and filled with Signa gel (D), and connected to the tDCS device (E). A conductivity check was performed (F) to make sure the impedance was below the safety threshold of 5 kΩ. Starting from 6 minutes after gel application, 2 mA tDCS was applied for 10 s every 2 min for a period of 1 h. After every stimulus of 10 s, subjects assessed the stimulation sensation and reported on potential side effects. In this figure, the setup is illustrated for the BL10 condition.

The protocol is shown in detail in Figure 2. At the start of each session the skin was cleaned with alcohol using a gauze pad. Next, the locations of the stimulation electrodes were indicated on the skin. Then, 4.8 ml gel (BL10, EMLA or placebo) was applied on the scalp underneath the stimulation electrodes over a surface area of approximately 25 cm^2^. Next, the tDCS cup electrodes were positioned as described above and filled with Signa gel. The cup electrodes were then connected to the tDCS device using standard Ag/AgCl ring electrodes and cables. Before tDCS was started, an impedance check was performed using the tDCS device. In case the impedance was below 5 kΩ, data collection stated 6 min after gel application. In case the impedance was > 5 kΩ, stimulation could not be started for safety reasons and standardization. The timepoints for which no data could be collected were treated as missing data. 2 mA tDCS was applied for 10 s every 2 min for a period of 1 h. Immediately after every 10-s stimulus, subjects assessed the tDCS stimulation sensation using a visual analog scale (VAS) rated between 0 (no sensation) and 10 (painful).

#### Side effects

In 9 of the 18 subjects side effect data was collected. Occurrence of the following side effects was assessed after each stimulus: tingling sensation, metal taste, headaches, dizziness, nausea, phosphenes, burning sensation and itchiness. For each side effect, subjects reported on the intensity of the side effect by indicating a score: 0 (side effect is not present), 1 (side effect is very slightly present), 2 (side effect is slightly present) or 3 (side effect is present). The occurrence of side effects was analyzed for each stimulation condition separately. For each time point (28 in total) and each side effect, the average side effect score was calculated across all subjects. In addition, for each side effect, an overall score was calculated by summing the scores across all time points for all subjects. This score was then expressed as a percentage of the maximum possible score 3 × 28 = 84 by dividing it by 84. For example, if a participant indicated 10 times a score of 1 for tingling sensation, 5 times the score 2 and 0 for the remaining 13 timepoints, the mean score for tingling sensation for this participant was calculated as 10 × 1 + 5 × 2 + 13 × 0 divided by 84 is 24%. The mean overall percentage across all subjects was then calculated for each side effect.

#### Statistical analysis

The VAS stimulation sensations were analyzed using a linear mixed model VAS ~ −1 + Gel + Time + Gel : Time + (1 | Subject) with fixed variables ‘Gel' and ‘Time' and, random variable ‘Subject', followed by a *post-hoc* analysis.

In the *post-hoc* analysis two-sided Wilcoxon signed rank tests at significance level 0.05 were used to compare the gels at three timepoints after gel application: 8, 30, and 60 min, chosen to be representative of the beginning, middle and end of the experiment. All reported *p*-values are Bonferroni corrected for three multiple comparisons. The effect sizes were calculated as the standardized mean difference using the Cohen's d formula for paired comparisons. According to Cohen and Sawilowsky, a Cohen's d value of 0.01 indicates a very small effect, 0.2 a small effect, 0.5 a medium effect, 0.8 a large effect, 1.2 a very large effect and 2 a huge effect (32). Confidence intervals shown in the VAS plot were calculated as 95% of the standard deviation divided by the square root of the number of subjects. All statistics were performed in MATLAB R2019b (MathWorks, Natick, MA, USA).

### Experiment 2

In experiment 2, the effect of a 2 mA DC current on the gel's resistivity and temperature was investigated for each gel: EMLA, BL10 and placebo. Additionally, we performed the same measurements on Signa gel, a highly conductive electrolyte gel designed to conduct electric signals in ECG recordings, amongst other applications.

#### Protocol

For each gel, a 2 mA (± 4 μA) DC current (I) of was applied using a NeuroConn DC-stimulator over a gel-filled cylinder with surface area (A) 4.91 cm^2^ and height (L) 2.5 cm for a duration of 1 h. The voltage (V) over the gel was measured using a National Instruments DAQ NI-USB 6364 (National Instruments, Austin, Texas, USA) with a sample frequency of fs = 1,024 Hz. The resistance (R) was calculated using Ohm's Law (V = I x R), from which the resistivity (ρ) was calculated using formula ρ = R x (A / L). Temperature was measured in the center of the cylinder using a Fluke Volt thermocouple (Everett, Washington, USA) with a precision of 0.5 °C and a sample frequency fs = 2 Hz.

## Results

### Experiment 1

#### Effect of topical anesthetic gels on tDCS stimulation sensation

**Figure 3:**
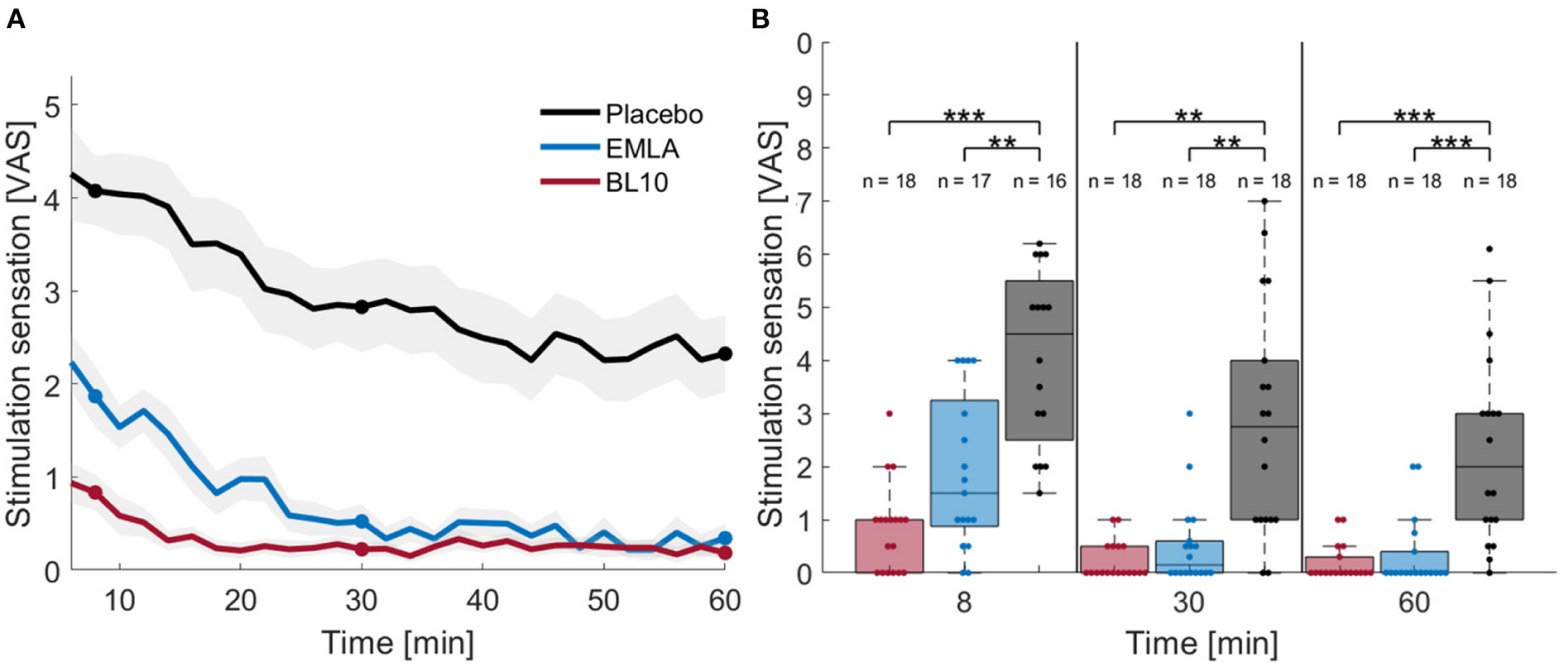
Effect of topical anesthetic gels on tDCS stimulation sensation. **(A)** The mean stimulation sensations in time for the BL10 gel (red), EMLA (blue) and placebo gel (black), with confidence intervals shown in gray. Subjects indicated significantly higher stimulation sensations for the placebo gel, compared to the gels containing anesthetics. **(B)** The results of the *post-hoc* analysis at the three timepoints 8, 30 and 60 min after gel application indicated significant differences between the gels containing anesthetics compared to placebo with **p < 0.01, ***p < 0.001.

Figure 3 shows the mean stimulation sensations at group level for the three gels over time (A) and at three specific timepoints: 8, 30, and 60 min after gel application (B). In general, subjects indicated higher stimulation sensations for the placebo gel compared to the gels containing anesthetics. For the placebo gel, subjects indicated an average stimulation sensation of 4.1 ± 1.7 on the VAS 8 min after gel application. The sensation decreased slightly to 2.8 ± 2.2 at 30 min and to 2.3 ± 1.8 after 1 h. For EMLA gel, sensations were on average 1.9 ± 1.5 at 8 min after gel application. Sensations decreased to 0.5 ± 0.8 at 30 min and to 0.3 ± 0.7 after 1 h. For the BL10 gel, subjects immediately rated low stimulation sensations. Eight min after gel application, subjects indicated 0.8 ± 0.8 on the VAS. The sensations decreased to 0.2 ± 0.4 at 30 min and remained at 0.2 ± 0.3 after 1 h.

The linear mixed model indicated a significant effect of the gels on the VAS stimulation sensation (*p* < 0.0001) and a significant interaction effect between gel and time (*p* < 0.0001). The *post-hoc* analysis at 8 min after gel application showed that the sensation for both the BL10 and EMLA gels were significantly lower compared to the placebo gel (*p* = 0.0004, Cohen's d = 1.66 and *p* = 0.006, Cohen's d = 0.96 respectively). In addition, there was a trend for the BL10 gel to give lower VAS sensations compared to EMLA but this trend was not significant after Bonferroni correction (*p* = 0.079, Cohen's d = 0.68). After 30 min, the BL10 and EMLA gel sensations remained significantly lower than the placebo sensations (p = 0.002, Cohen's d = 1.16 and p = 0.0013, Cohen's d = 1.23 respectively), with the BL10 and EMLA sensations not significantly different (*p* = 0.8965, Cohen's d = 0.3094). One hour after gel application, subjects still indicated significantly lower stimulation sensation for the BL10 and EMLA gel (*p* = 0.0004, Cohen's d = 1.1443 and p = 0.0007, Cohen's d = 1.0277 respectively) and while BL10 and EMLA remained similar (*p* = 0.4609, Cohen's d = 0.24).

#### Effect of topical anesthetic gels on tDCS side effects

**Figure 4:**
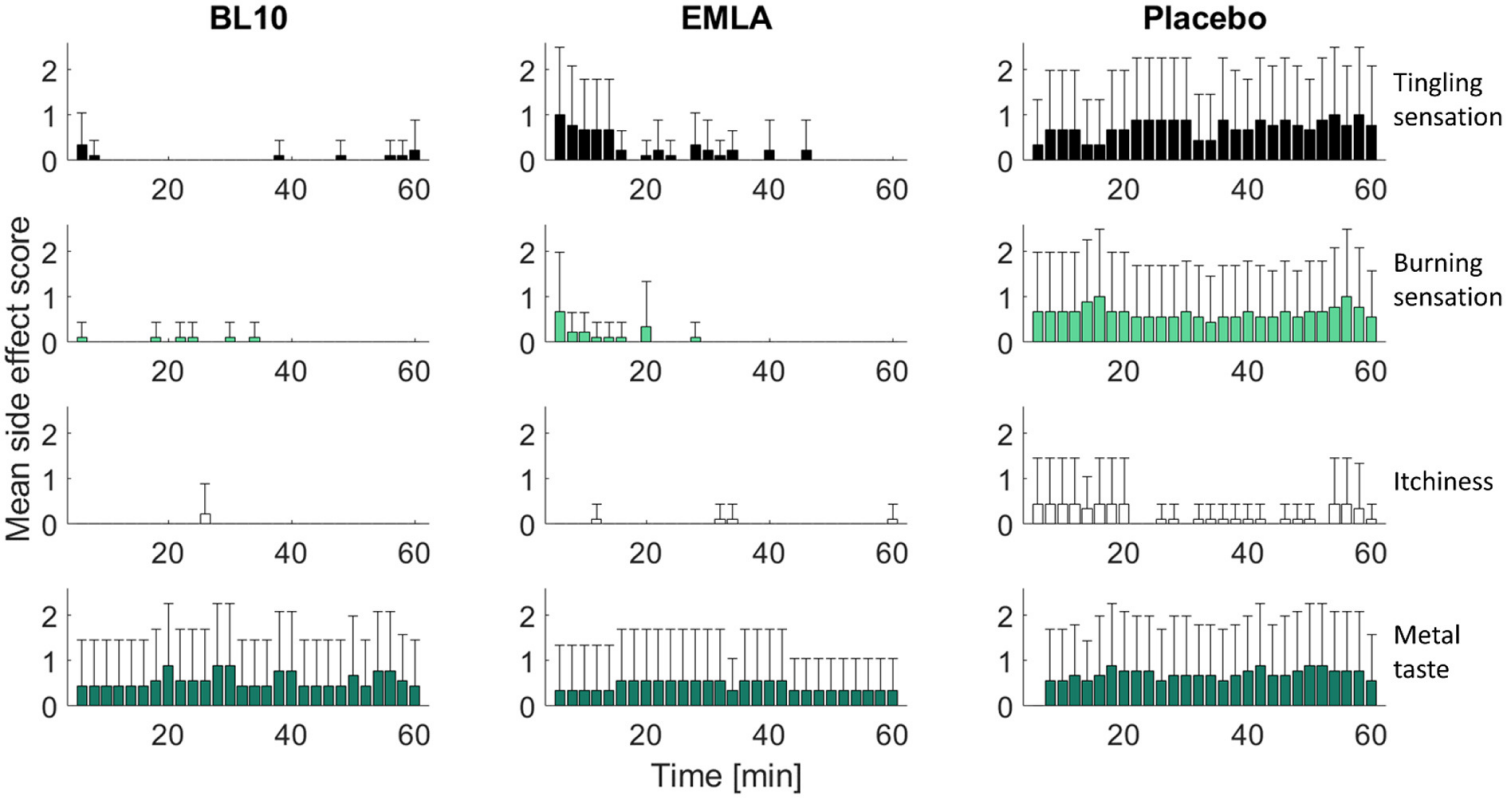
Effect of topical anesthetic gels on tDCS side effects. The side effects are shown as the mean side effect scores for nine subjects in time with the error bars indicating the standard deviation. In general, subjects experienced less side effects for the BL10 and EMLA gel compared to placebo. For the placebo gel, the most commonly reported side effects were: tingling sensation, burning sensation, itchiness and metal taste while other side effects were reported much less frequently. For the BL10 and EMLA gel, tingling sensation, burning sensation and itchiness were reduced compared to placebo, while the scores for metal taste were not influenced by the anesthetic gels. Although both anesthetic gels reduced sensations in the scalp, the sensations for the EMLA gel remained relatively high in the beginning of the experiment compared to the BL10 gel. An overview of all side effects is given in the Supplementary Results.

In general, subjects experienced few side effects, see Figure 4. In the majority of the surveyed sessions, subjects indicated they experienced only one or two side effects. In only five sessions, subjects reported they experienced more than two side effects, while in four sessions subjects indicated they experienced no side effects at all. The most commonly reported side effects were: tingling sensation, burning sensation, itchiness and metal taste. Other side effects were reported much less frequently.

In general, subjects experienced less side effects for the BL10 and EMLA gel compared to placebo. For both anesthetic gels, side effects that are related to sensations in the scalp, such as tingling sensation, burning sensation and itchiness, were reported much less frequently compared to the placebo gel. For the BL10 gel, the side effects that are related to sensations in the scalp were reduced immediately after gel application. In case of the EMLA gel, the side effect scores for sensations in the scalp were also reduced but only toward the middle of the experiment. In the beginning of the experiment, the side effect scores were similar to the scores of the placebo gel.

The mean overall side effect percentage for tingling sensation was 24% for placebo, while it was only 7% for both anesthetic gels. For burning sensation, the reduction was even more profound. For placebo, the mean overall side effect percentage was 22%, while it was only 1% and 2% for BL10 and EMLA gel respectively. For itchiness the mean overall side effect percentage for placebo of 7% was reduced to >1% and 1% for BL10 and EMLA gel respectively. Side effects that are related to co-stimulation of other facial nerves than the nerves that innervate the scalp, such as the occipital and trigeminal nerves, were not affected by the anesthetic gels. For example, the side effect scores for metal taste were similar for all gels: 19% for BL10 gel, 15% for EMLA and 23% for placebo. An overview of all side effects is given in the Supplementary Results.

### Experiment 2

#### Effect of DC current on gel resistivity and temperature

**Figure 5:**
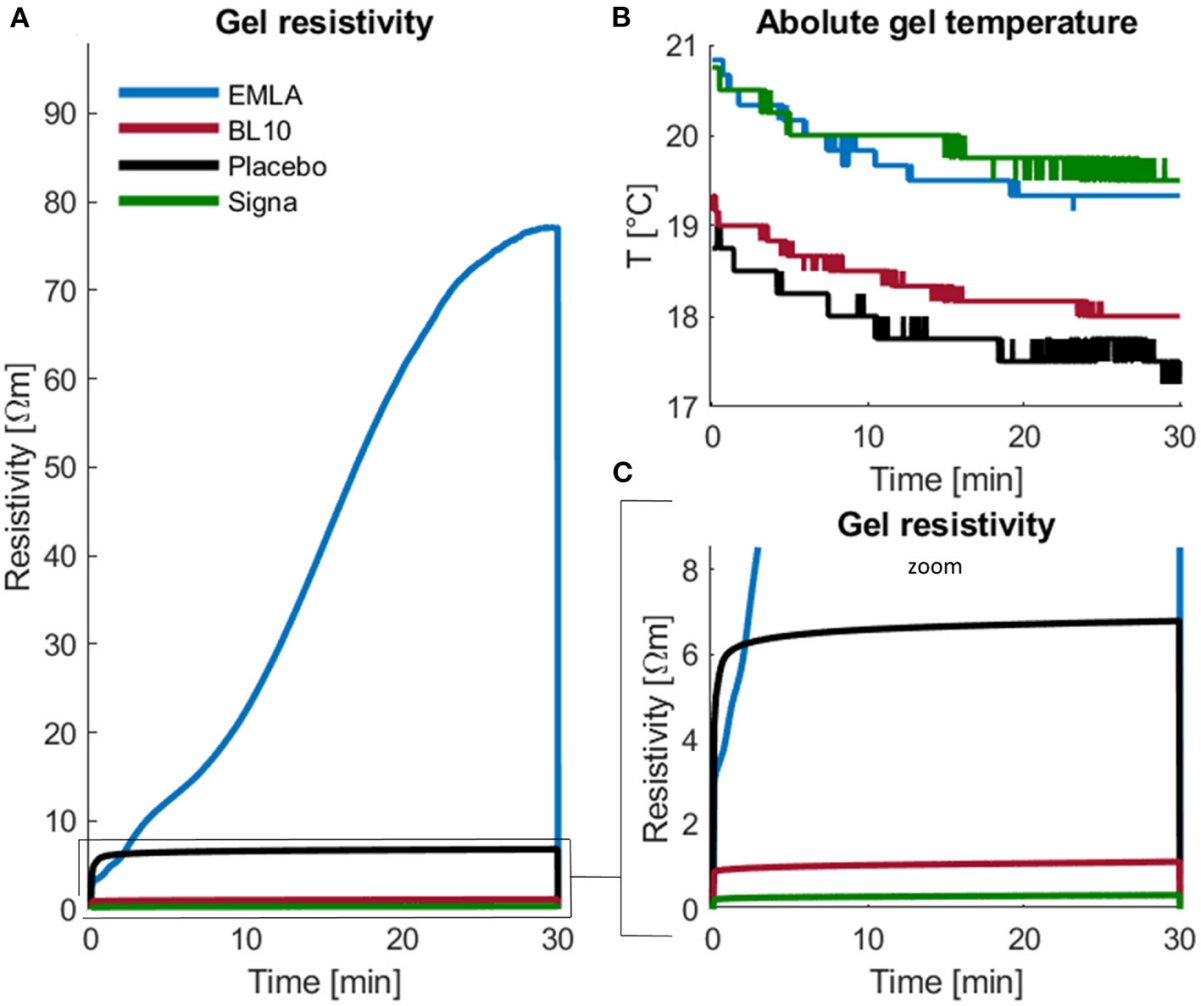
Effect of DC current on gel resistivity and temperature. In an *in-vitro* experiment, a DC current of 2 mA was applied over samples of the BL10 gel (red), EMLA gel (blue), placebo gel (black), and Signa gel (green) for 30 min. **(A)** Gel resistivity was relatively stable for all gels except for the EMLA gel, which showed a larger increase. **(C)** The resistivity of the BL10 gel was similar to the resistivity of the conductive Signa gel, whereas the resistivity of the placebo gel was slightly higher. **(B)** Temperature measurements showed that the gel temperature decreased steadily over time to room temperature. The illustrated results are the calculated average of three independent measurements, see Supplementary Results.

The *in vitro* measurements of gel conductivity and temperature revealed an odd gel property of the EMLA gel. When a current of 2 mA was applied over the BL10 gel, the resistivity remained stable at ρ = 1.1 Ωm for at least 1 h, see Figure 5. Similarly, the resistivity of the placebo gel remained stable at ρ = 6.8 Ωm. These resistivities of the BL10 and placebo gel are in the same order of magnitude as the resistivity of the Signa gel, which remained stable at ρ = 0.3 Ωm. Yet, the resistivity of the EMLA gel increased extremely up to ρ = 77.1 Ωm after 1 h of DC stimulation. Although the EMLA resistivity increased strongly, the temperature measurements did not show a notable difference compared to the other gels. For all gels, the temperature showed a small steady decreased to room temperature.

## Discussion

We validated the use of an optimized compounded anesthetic gel for the development of a novel tDCS control condition in which peripheral nerve co-stimulation is blocked during tDCS. Our results showed that topical anesthetics can successfully reduce peripheral co-stimulation sensations during stimulation: subjects indicated lower stimulation sensations on the VAS for both anesthetics gels, BL10 and EMLA, compared to the placebo gel (Figure 3). The tDCS side effect analysis showed similar results with subjects indicating they experienced tingling or burning sensations during tDCS for the placebo gel condition but experienced far less side effects related to sensation in the scalp for the anesthetic gels (Figure 4).

Recent literature has highlighted the potential confound of peripheral nerve co-stimulation during tDCS experiments. Vannest et al. (2) showed that some tDCS effects on memory appear to be driven by co-stimulation of the occipital nerve. They suggested the neuromodulatory effect is caused by activation of the noradrenergic pathway due to co-stimulation of the occipital nerve. This potential noradrenergic mechanism caused by cranial or cervical nerve co-stimulation during tDCS is discussed in detail in a recent opinion piece by van Boekholdt et al. (14). Adair et al. (19) also recently reviewed the literature on cranial nerve stimulation and emphasized that stimulation of these nerves is inevitable when tDCS is applied using stimulation electrodes on the scalp. Our results indicated topical anesthetics can be used to blocks peripheral nerve co-stimulation and allow the development of a novel tDCS control condition that could allow researchers to investigate whether the observed neuromodulatory effects during tDCS are caused by direct stimulation of the brain or by co-stimulation of peripheral nerves in the scalp.

### Effectiveness of topical anesthetic gels at reducing stimulation sensations

Both anesthetic gels significantly lowered the stimulation sensation indicated on the VAS. The Cohen's d values indicated large to huge effect sizes when comparing the VAS scores for the anesthetic gels to the placebo gel at the three timepoints. Interestingly, the BL10 gel appeared to act more quickly than EMLA gel. However, this trend did not reach significance (*p* = 0. 0791) after Bonferroni's multiple comparison correction, although the Cohen's d value showed a medium to large effect. The lack of significance might be due to the relatively high variability that is often present in subjective measures in human research. Nevertheless, it is interesting to point out a few reasons why the BL10 may act more quickly than the EMLA gel. Firstly, the BL10 gel contains benzocaine which is expected to diffuse faster through the cell membrane according to its dissociation constant (pKa _benzocaine_ = 3.5) compared to lidocaine and prilocaine in the EMLA gel. Secondly, the BL10 gel contains double the concentration of anesthetics compared to the EMLA gel. Lastly, the BL10 is water-based. A water-based gel can contain higher concentrations of anesthetics due to the higher solubility of the protonated form of the anesthetics, administered as a hydrochloride salt, in water compared to the oily EMLA gel. In addition, the water-based gel can penetrate faster into the skin than the dense oily EMLA gel.

### Effect of topical anesthetics on tDCS side effects

In our study, the most commonly reported side effects during tDCS with the placebo gel were: tingling sensation, burning sensation, itchiness and metal taste. A systematic review on adverse effects associated with tDCS (33) indicated itchiness as the main side effect, followed by tingling sensation, headache and burning sensation. Compared to these findings, subjects in our study reported burning sensations more frequently. This might be due to our relatively high stimulation amplitude of 2 mA compare to the more commonly used amplitude of 1 mA. Our results also indicated that the application of topical anesthetics under the stimulation electrodes greatly reduced tingling sensations, burning sensations and itchiness caused by co-stimulation of peripheral nerves in the scalp. The applied anesthetics block neural signal transduction in those peripheral nerves and thereby reduce tDCS side effects related to co-stimulation. Interestingly, the anesthetics appeared to have little effect on side effect scores for metal taste, a result of co-stimulation of gustatory nerves such as the glossopharyngeal nerve and the vagus nerve which innervate the tongue. One would not expect a topical anesthetic applied on the scalp to have an effect on those nerves, which is consistent with our findings. In brief, topical anesthetics applied on the scalp under the stimulation electrodes appear to reduce tDCS side effects that are relate to sensations in the scalp, but do not affect other side effects.

### Effect of a DC current on gel resistivity and temperature

In an *in vitro* experiment, we investigated the effect of a 2 mA DC current on two important gel properties: gel temperature and gel resistivity. Our temperature measurements showed that a direct current of 2 mA does not cause heating for at least 30 min of stimulation. In fact, we observed a slight decrease in temperature probably due to the gel cooling down to room temperature rather than an effect of the DC current. The resistivity measurements showed that the resistivity of the BL10 and the placebo gel remained stable and relatively low within the same order of magnitude as the highly conductive Signa gel. Compared to the placebo gel, the resistivity of the BL10 gel was approximately six times lower. The higher conductivity of the BL10 gel complies with the expectations because the BL10 gel is based on the same carbomer gel as the placebo gel but contains additional charged molecules such as the protonated anesthetic molecules. The EMLA gel, however, showed a large increase in resistivity compared to the other gels. This undesired gel property of EMLA makes it a poor choice for use in human tDCS experiments as these changes in resistivity could lead to unstable impedances during stimulation and may cause additional or more profound side effects. For example, more phosphenes were reported for the EMLA gel compared to the BL10 and placebo (Supplementary Figure S1).

### Safety of topical anesthetics

Topical anesthetics are considered safe to be used (30), however some side effects may occur such as headaches, dizziness, itchiness, skin swelling or skin irritation (34). It is important to use local anesthetics according to guidelines (35). If not used properly, toxic side effects can occur as a result of either an allergic reaction, high systemic exposure due to overdosing or interactions with other pharmacological agents (36). These toxic effects may lead to serious adverse events such as: confusion, tightness in the chest, loss of consciousness, seizure, respiratory depression, vasodilation or cardiac arrest (37). A small number of cases have been reported in which serious toxic effects occurred after the application of topical anesthetics (38, 39), which resulted in death in some cases (37, 40, 41). Hence, when considering the use of topical anesthetics, it is important to use it according to guidelines and be aware of the potential risks.

### Efficacy of anesthetics

Although the topical anesthetics significantly reduced tDCS stimulation sensations, they were not always able to completely block peripheral nerve input. More invasive interventions would be needed to ensure complete blockage. In animal research, for example, one can simply cut the nerves in the scalp or apply stimulation underneath the scalp directly on the skull. Considering the trade-off between efficacy and invasiveness, the use of topical anesthetics seems to be the optimal solution. However, it is also important to address the issue of adding a pharmacological agent in neuromodulation experiments. Adding anesthetics to a tDCS stimulation condition also includes adding potential side effects that are not related to the stimulation. Despite this, our results showed that the novel tDCS stimulation condition in which topical anesthetics are applied underneath the stimulation electrodes, were able to sufficiently block peripheral co-stimulation during tDCS and has the potential to be used in tDCS research to isolate direct brain effects from peripheral effects.

## Conclusion

We have shown that topical anesthetic gels can be used to block peripheral nerve co-stimulation during tDCS. Our newly developed BL10 gel showed a tendency to work faster than EMLA gel, however this trend did not reach significance. In addition, the *in vitro* experiment revealed that the EMLA gel is unstable in terms of resistivity when a DC current is applied over the gel, while the resistivity of the BL10 gel remained stable. These findings suggest that the BL10 gel is the optimal topical anesthetic to block peripheral nerve co-stimulation in tDCS experiments. We suggest to use this novel tDCS stimulation condition, in which peripheral co-stimulation is blocked using the BL10 gel, as an additional control condition in tDCS research. This additional control condition would allow neuroscientists to separate neuromodulatory effects caused by the electric field in the brain from peripheral effects due to nerve co-stimulation in the scalp. More insight in the underlying neurophysiological mechanisms of tDCS will ensure the development of more effective applications of this versatile neuromodulation technique.

## Data availability statement

The raw data supporting the conclusions of this article will be made available by the authors, without undue reservation.

## Ethics statement

The study involving human subjects was reviewed and approved by the UZ/KU Leuven Research Ethical Committee (S63709). The subjects provided their written informed consent to participate in this study.

## Author contributions

SK designed the experiments, acquired and analyzed the data, and wrote the article. J-JO and MMcL contributed by discussing and editing the experimental design and reviewing the manuscript. All authors contributed to the article and approved the submitted version.

## Funding

This work was supported by the following grants: FWO SB fellowship 1S32421N, FWO project G0B4520N, and NIH grant 1R01MH123508-01.

## Conflict of interest

The authors declare that the research was conducted in the absence of any commercial or financial relationships that could be construed as a potential conflict of interest.

## Publisher's note

All claims expressed in this article are solely those of the authors and do not necessarily represent those of their affiliated organizations, or those of the publisher, the editors and the reviewers. Any product that may be evaluated in this article, or claim that may be made by its manufacturer, is not guaranteed or endorsed by the publisher.

## References

[1] Yamada Y Sumiyoshi T. Neurobiological mechanisms of transcranial direct current stimulation for psychiatric disorders; neurophysiological, chemical, and anatomical considerations. Front Hum Neurosci. (2021) 15:1–10. 10.3389/fnhum.2021.631838 33613218 PMC7890188

[2] Vanneste S Mohan A Yoo HB Huang Y Luckey AM McLeod SL. The peripheral effect of direct current stimulation on brain circuits involving memory. Sci Adv. (2020) 6:1–20. 10.1126/sciadv.aax9538 33148657 PMC7673706

[3] Savic B Meier B. How transcranial direct current stimulation can modulate implicit motor sequence learning and consolidation: a brief review. Front Hum Neurosci. (2016) 10:26. 10.3389/fnhum.2016.00026 26903837 PMC4748051

[4] Ke Y Wang N Du J Kong L Liu S Xu M. The effects of transcranial direct current stimulation (tDCS) on working memory training in healthy young adults. Front Hum Neurosci. (2019) 13:1–10. 10.3389/fnhum.2019.00019 30774590 PMC6367257

[5] Reis J Schambra HM Cohen LG Buch ER Fritsch B Zarahn E. Noninvasive cortical stimulation enhances motor skill acquisition over multiple days through an effect on consolidation. Proc Natl Acad Sci U S A. (2009) 106:1590–5. 10.1073/pnas.0805413106 19164589 PMC2635787

[6] Kang EK Paik NJ. Effect of a tDCS electrode montage on implicit motor sequence learning in healthy subjects. Exp Transl Stroke Med. (2011) 3:2–7. 10.1186/2040-7378-3-4 21496317 PMC3101127

[7] Goodwill AM Reynolds J Daly RM Kidgell DJ. Formation of cortical plasticity in older adults following tDCS and motor training. Front Aging Neurosci. (2013) 5:1–9. 10.3389/fnagi.2013.00087 24367333 PMC3854104

[8] Bashir S Al-Hussain F Hamza A Asim Niaz T Albaradie R Habib SS. Cognitive function assessment during 2 mA transcranial direct current stimulation in DLPFC in healthy volunteers. Physiol Rep. (2019) 7:1–7. 10.14814/phy2.14264 31660693 PMC6817993

[9] Lüdemann-Podubecká J Bösl K Rothhardt S Verheyden G Nowak DA. Transcranial direct current stimulation for motor recovery of upper limb function after stroke. Neurosci Biobehav Rev. (2014) 47:245–59. 10.1016/j.neubiorev.2014.07.022 25108036

[10] Salling MC Martinez D. Brain stimulation in addiction. Neuropsychopharmacology. (2016) 41:2798–809. 10.1038/npp.2016.80 27240657 PMC5061891

[11] Shiozawa P Fregni F Benseñor IM Lotufo PA Berlim MT Daskalakis JZ . Transcranial direct current stimulation for major depression: an updated systematic review and meta-analysis. Int J Neuropsychopharmacol. (2014) 17:1443–52. 10.1017/S1461145714000418 24713139

[12] Nitsche MA Paulus W. Noninvasive brain stimulation protocols in the treatment of epilepsy: current state and perspectives. Neurotherapeutics. (2009) 6:244–50. 10.1016/j.nurt.2009.01.003 19332316 PMC5084200

[13] Majdi A van Boekholdt L Sadigh-Eteghad S Mc Laughlin M. A systematic review and meta-analysis of transcranial direct-current stimulation effects on cognitive function in patients with Alzheimer's disease. Mol Psychiatry. (2022) 27:2000–9. 10.1038/s41380-022-01444-7 35115703

[14] van Boekholdt L Kerstens S Khatoun A Asamoah B Mc Laughlin M. tDCS peripheral nerve stimulation: a neglected mode of action? Mol Psychiatry. (2021) 26:456–61. 10.1038/s41380-020-00962-6 33299136

[15] Nitsche M Nitsche M Paulus W Paulus W. Excitability changes induced in the human motor cortex by weak transcranial direct current stimulation. J Physiol. (2000) 3 (527 Pt):633–9. 10.1111/j.1469-7793.2000.t01-1-00633.x 10990547 PMC2270099

[16] Rahman A Reato D Arlotti M Gasca F Datta A Parra LC. Cellular effects of acute direct current stimulation: somatic and synaptic terminal effects. J Physiol. (2013) 591:2563–78. 10.1113/jphysiol.2012.247171 23478132 PMC3678043

[17] Asamoah B Khatoun A Mc Laughlin M. tACS motor system effects can be caused by transcutaneous stimulation of peripheral nerves. Nat Commun. (2019) 10:266. 10.1038/s41467-018-08183-w 30655523 PMC6336776

[18] Rampersad SM Janssen AM Lucka F Aydin Ü Lanfer B Lew S. Simulating transcranial direct current stimulation with a detailed anisotropic human head model. IEEE Trans Neural Syst Rehabil Eng. (2014) 22:441–52. 10.1109/TNSRE.2014.2308997 24760939

[19] Adair D Truong D Esmaeilpour Z Gebodh N Borges H Ho L. Electrical stimulation of cranial nerves in cognition and disease. Brain Stimul. (2020) 13:717–50. 10.1016/j.brs.2020.02.019 32289703 PMC7196013

[20] Kessler SK Turkeltaub PE Benson JG Hamilton RH. Differences in the experience of active and sham transcranial direct current stimulation. Brain Stimul. (2012) 5:155–62. 10.1016/j.brs.2011.02.007 22037128 PMC3270148

[21] Fanselow EE. Central mechanisms of cranial nerve stimulation for epilepsy. Surg Neurol Int. (2012) 3:S247–54. 10.4103/2152-7806.103014 23230529 PMC3514917

[22] Mercante B Ginatempo F Manca A Melis F Enrico P Deriu F. Anatomo-Physiologic Basis for Auricular Stimulation Med Acupunct. (2018) 30:141–50. 10.1089/acu.2017.1254 29937968 PMC6011382

[23] Couto LB Moroni CR dos Reis Ferreira CM Elias-Filho DH Parada CA Pelá IR. Descriptive and functional neuroanatomy of locus coeruleus-noradrenaline-containing neurons involvement in bradykinin-induced antinociception on principal sensory trigeminal nucleus. J Chem Neuroanat. (2006) 32:28–45. 10.1016/j.jchemneu.2006.03.003 16678997

[24] Aston-Jones G Shipley MT Chouvet G Ennis M van Bockstaele E Pieribone V. Afferent regulation of locus coeruleus neurons: anatomy, physiology and pharmacology. Prog Brain Res. (1991) 88:47–75. 10.1016/S0079-6123(08)63799-1 1687622

[25] Aston-Jones G Rajkowski J Cohen J. Role of locus coeruleus in attention and behavioral flexibility. Biol Psychiatry. (1999) 46:1309–20. 10.1016/S0006-3223(99)00140-7 10560036

[26] Berridge CW Waterhouse BD. The locus coeruleus-noradrenergic system: modulation of behavioral state and state-dependent cognitive processes. Brain Res Brain Res Rev. (2003) 42:33–84. 10.1016/S0165-0173(03)00143-7 12668290

[27] Sara SJ. The locus coeruleus and noradrenergic modulation of cognition. Nat Rev Neurosci. (2009) 10:211–23. 10.1038/nrn2573 19190638

[28] Fozzard HA Sheets MF Hanck DA. The sodium channel as a target for local anesthetic drugs. Front Pharmacol. (2011) 2:68. 10.3389/fphar.2011.00068 22053156 PMC3205381

[29] Becker DE Reed KL. Essentials of local anesthetic pharmacology. Anesth Prog. (2006) 53:98–109. 10.2344/0003-3006(2006)53[98:EOLAP]2.0.CO;2 17175824 PMC1693664

[30] Malamed SF. Handbook of local anesthesia. In: Handbook of Local Anesthesia. St. Louis, MO: Elsevier (2013).

[31] Singh R Al Khalili Y. Benzocaine. In: StatPearls. Treasure Island, FL: StatPearls Publishing (2022).

[32] Sawilowsky SS. Very large and huge effect sizes. J Mod Appl Stat Methods. (2009) 8:597–9. 10.22237/jmasm/1257035100

[33] Brunoni AR Eduardo J Amadera D Berbel B. A systematic review on reporting and assessment of adverse effects associated with transcranial direct current stimulation. Int J Neuropsychopharmacol. (2011) 14:1133–45. 10.1017/S1461145710001690 21320389

[34] IBM Watson Health. Lidocaine (Topical Application Route). Mayo Foundation for Medical Education and Research. (2022). Available online at: https://www.mayoclinic.org/drugs-supplements/lidocaine-topical-application-route/side-effects/drg-20072776 (accessed April 13, 2022).

[35] Weaver JM. Calculating the maximum recommended dose of local anesthetic. J Calif Dent Assoc. (2007) 35:61–3. 17269289

[36] Bahar E Yoon H. Lidocaine: a local anesthetic, its adverse effects and management. Medicina (Kaunas). (2021) 57:782. 10.3390/medicina57080782 34440986 PMC8399637

[37] Malamed SF. Morbidity, mortality and local anaesthesia. Prim Dent Care. (1999) 6:11–5. 10752458

[38] Mehra P Caiazzo A Maloney P. Lidocaine toxicity. Anesth Prog. (1998) 45:38–41. 9790008 PMC2148953

[39] Marra DE Yip D Fincher EF Moy RL. Systemic toxicity from topically applied lidocaine in conjunction with fractional photothermolysis. Arch Dermatol. (2006) 142:1024–6. 10.1001/archderm.142.8.1024 16924052

[40] Hersh EV Helpin ML Evans OB. Local anesthetic mortality: report of case. ASDC J Dent Child. (1991) 58:489–91. 1783701

[41] Young D. Student's death sparks concerns about compounded preparations. Am J Heal Pharm. (2015) 62:450–2. 10.1093/ajhp/62.5.450 15745900

