# Supplementary material for: A novel tDCS control condition using optimized anesthetic gel to block peripheral nerve input
Source: Front Neurol. 2022 Nov 14;13:1049409. doi: 10.3389/fneur.2022.1049409 (PMC9702085; doi:10.3389/fneur.2022.1049409)
Supplement: Supplementary file 1 [file Data_Sheet_1.pdf]

Supplementary results

tDCS side effects

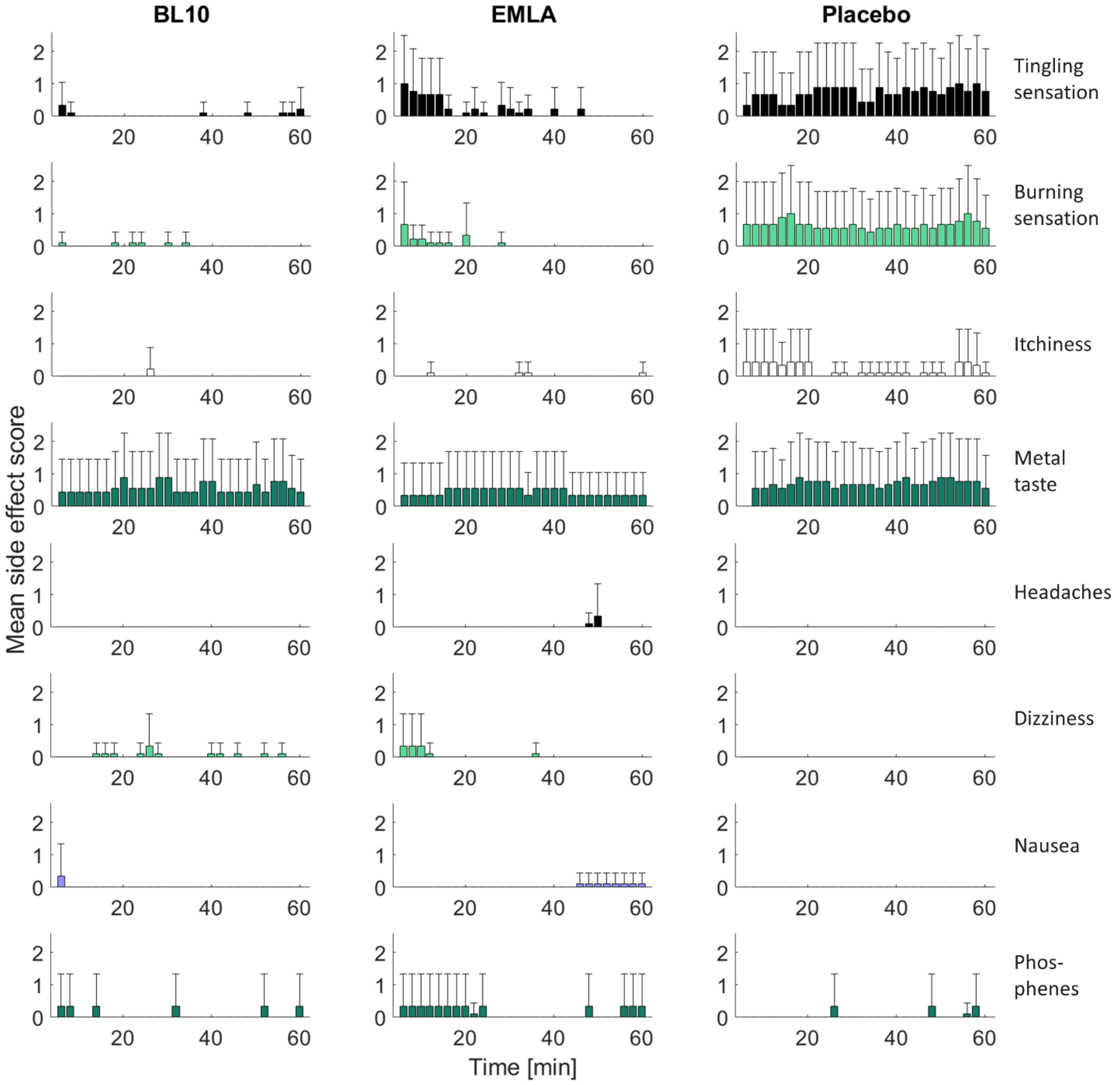

FIGURE S1

Effect of topical anesthetic gels on tDCS side effects. The side effects are shown as the average side effect scores for nine subjects with the error bars indicating the standard deviation. In general, subjects experienced less side effects for the BL10 and EMLA gel compared to placebo. For the placebo gel, the most commonly reported side effects were: tingling sensation, burning sensation, itchiness and metal taste, while other side effects were reported much less frequently. For the BL10 and EMLA gel, itchiness, tingling and burning sensations were reduced compared to placebo, while the scores for metal taste were not influenced by the anesthetic gels. Although both anesthetic gels reduced the sensation, the sensations for the EMLA gel remained relatively high compared to the BL10 gel in the beginning of the experiment. Other side effects were reported much less frequently.

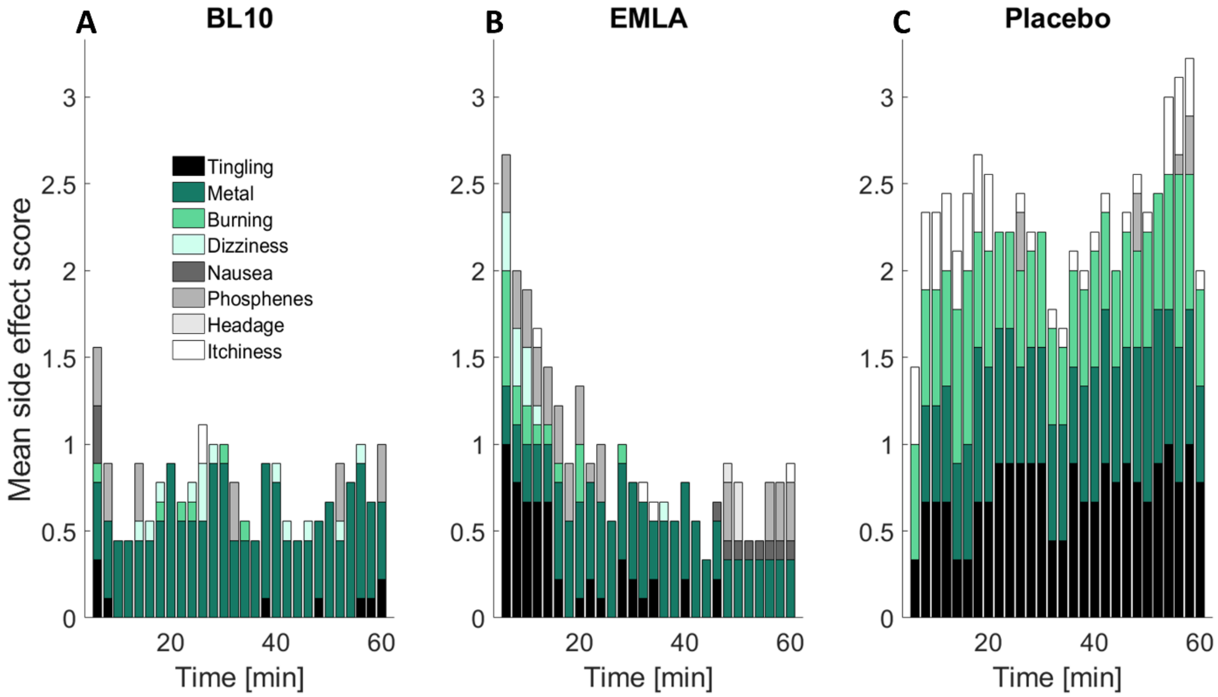

FIGURE S2

Sum of average side effect scores per stimulation condition. The side effects are shown as the sum of the average side effect scores for nine subjects. The most commonly reported side effects were: tingling sensation, itchiness, metal taste, and burning sensation while other side effects were reported much less frequently. In general, subjects experienced less side effects for the A) BL10 and B) EMLA gel compared to the C) placebo gel. Especially side effects that are related to sensations in the scalp, like tingling and burning sensations, were reduced by the topical anesthetics. For the BL10 gel, the tingling and burning sensations were reduced from the beginning of the experiment. In case of the EMLA gel, the sensations remained similar to the placebo scores in the beginning, but reduced to lower scores towards the middle of the session.

Conductivity and temperature measurements

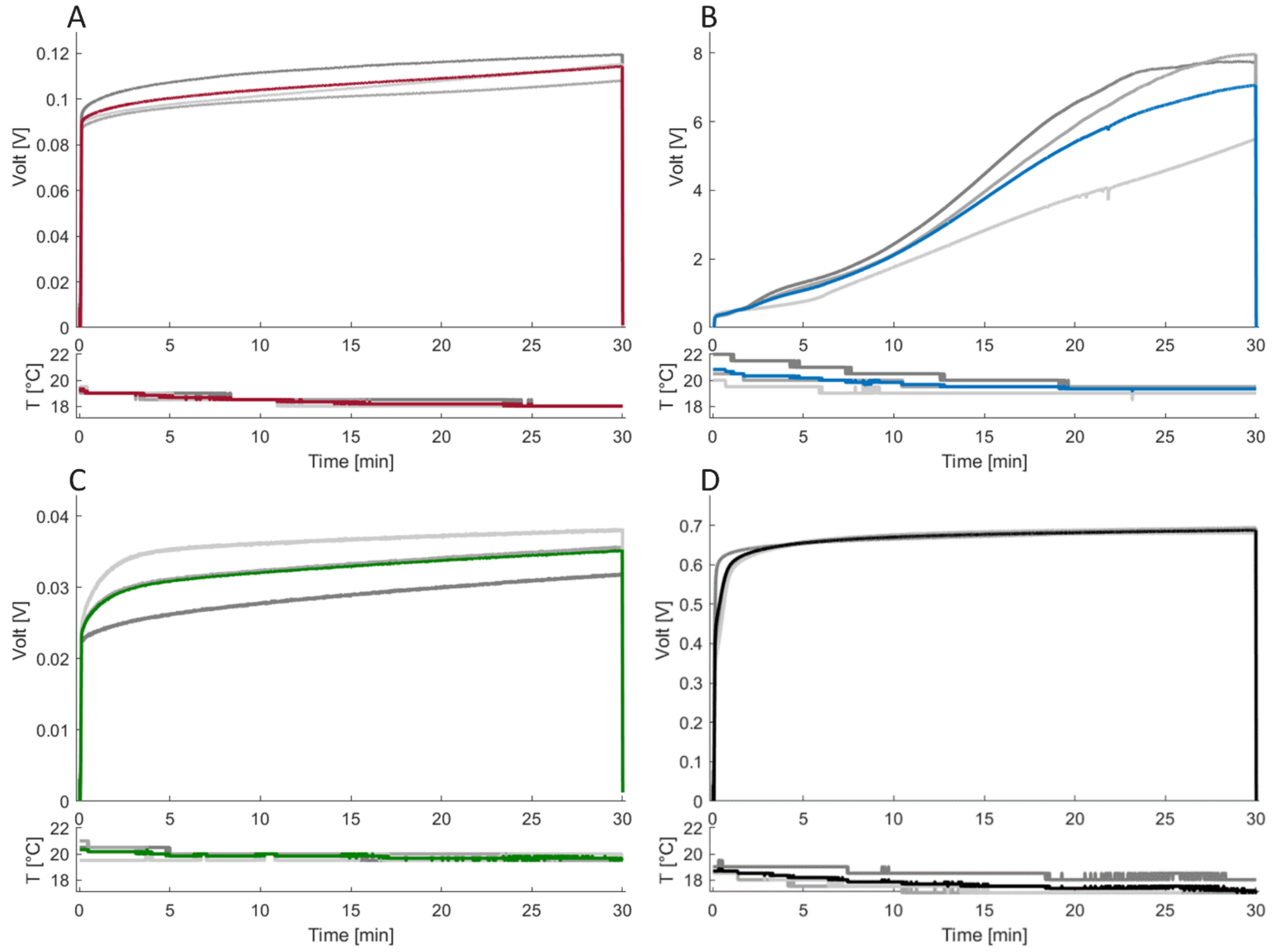

FIGURE S3

Effect of DC current on gel resistivity and temperature. In an in-vitro experiment, a DC current of 2 mA was applied over samples of the BL10 gel (A, red), EMLA gel (B, blue), Signa gel (C, green) and placebo gel (D, black), for 30 minutes. Gel resistivity was relatively stable for all gels except for the EMLA gel, which showed a larger increase (B). The resistivity of the BL10 gel (A) was similar to the resistivity of the conductive Signa gel (C), whereas the resistivity of the placebo gel was slightly higher (D). Temperature measurements showed that the gel temperature decreased steadily over time to room temperature. Each subfigure shows the three independent measurements in grey scale and the average in color.
